# Detection distances in desert dwelling, high duty cycle echolocators: A test of the foraging habitat hypothesis

**DOI:** 10.1371/journal.pone.0268138

**Published:** 2022-05-19

**Authors:** Nikita M. Finger, Marc Holderied, David S. Jacobs

**Affiliations:** 1 Department of Biological Sciences, University of Cape Town, Cape Town, South Africa; 2 School of Biological Sciences, University of Bristol, Bristol, United Kingdom; The University of Auckland - City Campus: University of Auckland, NEW ZEALAND

## Abstract

High Duty Cycle (HDC) echolocating bats use high frequency echolocation pulses that are clutter resistant, but their high frequencies give them limited range. Despite their unique ability to reject background clutter while simultaneously detecting fluttering prey, the frequency of their echolocation pulses has a strong correlation with level of environmental clutter, lower frequency pulses of HDC bats being associated with more open environments. The Foraging Habitat Hypothesis (FHH) proposes that the ecological significance of these lower frequency pulses in HDC bats in open environments is that they allow longer prey detection distances. To test the FHH, we compared the frequencies, Source Levels (SLs) and detection distances of *Rhinolophus capensis*, a HDC bat that has been shown to vary its call frequency in relation to habitat structure. As a further test of the FHH we investigated the SLs and detection distances of *Rhinolophus damarensis* (a heterospecific species that occurs in the same open desert environment as *R*. *capensis* but echolocates at a higher dominant pulse frequency). In the open desert, *R*. *capensis* emitted both lower frequency and higher SL pulses giving them longer detection distances than *R*. *capensis* in the cluttered fynbos. SL contributed more to differences in detection distances in both *R*. *capensis* and *R*. *damarensis* than frequency. In a few instances, *R*. *damarensis* achieved similar detection distances to desert–inhabiting *R*. *capensis* by emitting much higher SLs despite their average SLs being lower. These results suggest that lower frequency echolocation pulses are not a prerequisite for open desert living but may increase detection distance while avoiding energetic costs required for high SLs.

## Introduction

The success of bats as nocturnal hunters can be attributed to their use of echolocation [1,2]. The acoustic characteristics of their biosonar pulses play a crucial role in how bats detect, localize, and classify objects [3–8]. The distance over which bats can detect prey is determined by two key echolocation properties: pulse frequency and source level (SL) [9]. External properties of the environment, such as climate and habitat structure correlate with variation in pulse frequency [10–14]. One of the ways in which habitat structure influences echolocating bats is through clutter, the number of echoes produced by objects, e.g. vegetation, other than those from the target of interest. Variation in pulse frequency in responses to different levels of clutter has been observed in bats that use a clutter rejection echolocation system, i.e. High Duty Cycle (HDC) bats, despite their unique ability to reject background clutter and simultaneously detect fluttering prey [15–17]. For example, *Rhinolophus ferrumequinum* occupy a variety of different habitats with various levels of clutter/vegetation. In relatively open habitats this species emits lower echolocation frequencies [18].

The level of atmospheric attenuation a bat is subjected to in its habitat may impact the source level at which a bat emits its echolocation pulse. Atmospheric attenuation is a function of the frequency of the pulse; higher frequency pulses are attenuated more strongly [11,12,19]. An intricate interaction between pulse frequency, atmospheric attenuation, and the source levels at which bats emit their echolocation pulses could exist. Climate affects atmospheric attenuation, and hence echolocation, by a complex interaction between temperature and humidity amongst other climatic variables [11,12,19,20]. The interaction between these variables (i.e. climate, atmospheric attenuation, and a bats echolocation pulse parameters) can vary from arid to mesic habitats. Furthermore, differences in temperature and rainfall between arid and mesic habitats, are likely to be reflected in more open (less clutter) and denser vegetation (more clutter), respectively [12,20]. Bats, even those using clutter-rejecting HDC echolocation, foraging in low-clutter habitats would likely benefit from longer detection distances. Increased detection distances would allow them to search greater volumes of space more efficiently. The interaction between pulse frequency, atmospheric attenuation, source levels and clutter, is therefore crucial to understanding the ecological significance of differences in frequency and their resultant impact on detection distances in HDC bats.

The influence of habitat structure was formalized in the Foraging Habitat Hypothesis (FHH). The FHH proposes a close relationship between the range of echolocation frequencies used by bats and the degree of clutter they are exposed to in different habitats [10]. The FHH was first proposed for Low Duty Cycle (LDC) bats. In LDC bats the FHH proposes that bats increase pulse frequency in obstacle rich habitats for increased resolution of prey against background vegetation [10,21–23]. The ecological significance of the FHH in clutter rejection foragers (i.e. HDC bats) is less well established despite marked variation in echolocation pulse frequencies across biomes [18,24]. Unlike LDC bats, HDC bats have the unique ability to reject background clutter and simultaneously detect fluttering prey using high frequencies, Doppler Shift Compensation, and the acoustic glints generated from the wing beats of insects [15–17]. As a result, they can easily distinguish flying prey from background vegetation. Furthermore, as clutter specialists many of the smaller HDC bats species evolved the use of very high echolocation pulse frequencies allowing them to retrieve echoes from small objects (leaves, twigs, wings of insects) [25]. HDC bats place most of their acoustic energy into the second harmonic which is at a higher frequency than the fundamental [26]. At such high frequencies increases in resolution would be minimal because differences in frequency are not large enough to produce substantial differences in target strength [27]. Unlike LDC bats, observed differences in frequencies in HDC bats in environments characterized by different degrees of clutter cannot, therefore, be attributed to an increase in prey resolution in obstacle rich environments. The use of high frequencies subjects the echolocation pulses of these small HDC bats to extreme atmospheric attenuation resulting in shorter prey detection distances. In open environments these small bats would be unable to increase their flight speed to search the open space at a faster rate. This would potentially be disadvantageous because their high frequency pulses would cover a smaller volume of space. Despite this disadvantage, small HDC species occupy open environments such as deserts. However, they tend to use echolocation pulses of lower frequencies [18,24]. Such lower pulse frequencies may be associated with lower levels of clutter. If so, under the FHH modified for HDC bats, we propose that the lower frequency pulses used by HDC bats in open environments result in longer prey detection distances allowing them to search open space more efficiently. If so, differences in echolocation frequency between HDC bats foraging in open habitats and those foraging in more cluttered habitats should result in longer detection distances in the open habitats.

Detection distance is strongly affected by both frequency and SL [9]. Increasing pulse SL increases the distance over which that pulse can return echo information loud enough to be heard by the echolocating bat (i.e. the operational distance of echolocation) [9]. Technological advances in microphone array analysis have revealed that LDC bats, regardless of size, use extremely intense (high SL) echolocation pulses in the field [28–31]. However, the ecological significance of SL has only been explored in a few studies on LDC bats and these have shown its functional impact on prey detection distance. For example, Surlykke and Kalko [30] found that LDC bat species in the local tropical assemblage compensated for frequency-dependent attenuation by using higher SLs to achieve comparable detection distances. In contrast, two species in a temperate region with different foraging strategies and habitats (open air versus clutter) used a combination of frequency and SL to achieve appropriate but different detection distances in their respective habitats [32]. These findings suggest that both the physical properties of the habitat (i.e. vegetation structure, climate) and the specific ecology of the focal species must be considered when testing the FHH. Currently, the only information on SL and detection distances in HDC bats comes from laboratory studies [33–35]. Recent advances in cross-correlation methods (microphone array analysis) use the FM component of HDC bat pulses to accurately determine differences in arrival times of the pulses at each microphone in the array and therefore the position of the bat relative to the microphones. Using this method SLs of HDC bats can be calculated from the field and lab. Field studies are vital to understanding the natural SLs and resulting detection distances emitted by bats in their respective environments.

Our study tested the modified version of the FHH in two HDC species; *Rhinolophus capensis*, which occupies a range of arid and mesic habitats, and *R*. *damarensis* which occupies mainly arid habitats and is sympatric with an *R*. *capensis* population in the desert. *R*. *capensis* successfully inhabits a variety of biomes characterised by different degrees of clutter from fynbos (an evergreen, Mediterranean type shrubland that occurs in the eastern and western cape provinces of South Africa) to desert [24]. Odendaal *et al*. [24] found a positive correlation between level of clutter and frequency in these populations, with lower frequencies occurring at lower levels of clutter. Our study investigated two populations of *R*. *capensis*: *R*. *capensis* in the desert (echolocation frequency: 75 kHz) and *R*. *capensis* in the fynbos (echolocation frequency: 84 kHz) [24]. The desert population of *R*. *capensis* deviated from the correlation between pulse frequency and body size more than any of the other populations i.e. it had a lower pulse frequency than predicted by its body size [24]. Similarly, it also deviated more than the other populations from the relationship between pulse frequency and clutter [24]

*Rhinolophus damarensis*, inhabits the same desert as *R*. *capensis* and other less arid habitats, but uses an echolocation pulse frequency equivalent to *R*. *capensis* inhabiting the fynbos. The use of higher pulse frequencies in the desert should result in much shorter detection distances for *R*. *damarensis* than *R*. *capensis*. Given its similar size and wing loading to *R*. *capensis* [36], this would place *R*. *damarensis* at a considerable disadvantage because the volume of space within which they searched for prey could be greatly reduced in comparison. In addition to frequency, SL also influences detection distance [9,37,38]. For *R*. *damarensis* to achieve comparable detection distances to *R*. *capensis* in the desert they would need to use higher SLs (as was found for LDC bats) [30].

The FHH hypothesis proposes that differences between desert and fynbos *R*. *capensis* are the result of ecological selection for longer detection distances in desert bats in response to lower levels of clutter (increased volumes of space) in the desert [10]. If so, 1) detection distances for desert bats i.e. *R*. *capensis* and *R*. *damarensis* should be similar to each other but longer than fynbos *R*. *capensis* 2) SLs of *R*. *damarensis* should be higher than desert *R*. *capensis* if they are to have similar detection distances i.e. similar responses to open space given the differences in their pulse frequencies 3) differences in detection distances in *R*. *capensis* should largely be the result of differences in pulse frequency.

## Methods

### Study species

Our focal species is the endemic cape horseshoe bat, *Rhinolophus capensis*. *R*. *capensis*, is a medium sized bat weighing around 10 g– 16 g with a forearm length of 47 mm– 52 mm [27]. *R*. *capensis* forages in or near cluttered habitats [27,39]. Their diet consists mainly of Lepidoptera and Coleoptera [27]. *R*. *capensis* is largely restricted to the narrow coastal belt of the western and southern coast of South Africa between the coastline and the great escarpment encompassing a variety of different biomes [24]. Body size and echolocation frequency (up to 10 kHz gradual change across its range) vary across the geographic distribution of *R*. *capensis* [24,40]. Spatially defined mitochondrial groups do exist but there is gene flow across its range [24]. In addition, mitochondrial DNA structure revealed minimal genetic structure among different populations of *R*. *capensis* in Southern Africa indicating that the desert and fynbos populations are indeed the same species [24]. A study that used nuclear introns as markers for phylogenetic reconstruction also confirmed this [41]. *R*. *capensis* range is characterised by relatively open habitats in the North and West (desert and karoo biomes) spanning habitats characterised by an increase in clutter in the East (fynbos, albany thicket and forest) [24,42]. These differences in vegetation are the result of a latitudinal aridity gradient, aridity increasing northwards and longitudinal seasonality shift in rainfall [43,44]. South Africa is predominantly a summer rainfall region (with approximately half of *R*. *capensis* range occurring in these regions) with a seasonal shift in rainfall that is caused by the winter rainfall zone that occurs along the southwestern and southern tip of the African continent. The rainfall in the winter rainfall zone is predominantly caused by cold fronts from polar cyclone systems originating over the South Atlantic. The diminishing influence of these polar frontal systems in the north is correlated with a decrease in rainfall and an increase in aridity [43,44].

Despite gene flow in *R*. *capensis*, the marked differences in rainfall and vegetation across their range has resulted in a stable relationship between pulse frequency and increasing vegetation; pulse frequency increases from open areas in the Northwest (75 kHz) to highly cluttered habitats in the Southeast (86 kHz) [24]. Differences in body mass do not explain differences in resting frequency because populations with similar body mass have different echolocation frequencies. Similarly, relative humidity does not explain these substantial differences in pulse frequency [24].

In the north of their range, *R*. *capensis* co-occurs with *Rhinolophus damarensis* and in the south with *Rhinolophus clivosus* [24]. *R*. *damarensis* (forearm length: 47 mm—52 mm) and *R*. *clivosus* (forearm length: 56 mm– 57 mm) are both medium sized bats [27]. *R*. *capensis* echolocates at approximately 9 kHz lower than their respective heterospecifics in the northern and southern regions where they overlap with these two species. Variation in pulse frequency, body size, and the environment of *R*. *capensis*, coupled with the occurrence of sympatric congenerics in parts of their range provide an excellent opportunity for investigating factors that influence intraspecific phenotypic divergence.

### Sampling sites

Bats were recorded in two biomes with contrasting clutter levels (desert and fynbos) over the summer months (Desert: January–February 2017, Fynbos: March 2017). Levels of clutter can be estimated by satellite-based vegetation assessment using the metric Normalized Difference Vegetation Index (NDVI). The NDVI ranges from -1 to 1 with negative values corresponding to little or no vegetation cover and positive values corresponding to different degrees of vegetation cover [45]. The desert biome sampled in this study has a NDVI index of 0.22 and the fynbos has a NDVI index of 0.45 [24]. We recorded bats both close to day roosts (mix of commuting and foraging activity) and in foraging habitats. In the desert, *R*. *capensis* was recorded during emergence at Wondergat cave (a day roost) and at a nearby foraging area (dried riverbed) shortly after emergence. Foraging sites were identified by the presence of feeding buzzes in on-site recordings of bat echolocation pulses. The desert foraging site was located 3.9 km away from Wondergat cave (28°25’S 16°53’E) in the Lekkersing area. *R*. *damarensis* was recorded in the desert biome during emergence from Numeesberg mineshaft (28°26’S 17°01’E) and at the Orange River cave (28°42’S 17°32’E). Orange River cave was considered both a cave and foraging site because of the presence of feeding buzzes in the echolocation recordings of bats in this area. In the south coast, *R*. *capensis* was recorded at Hot Hole cave (34°27’S 20°26’E) in De Hoop Nature Reserve. Hot Hole cave was characterised as both cave and foraging site. Echolocation pulses recorded at Hot Hole cave were therefore grouped into an emergence/foraging category because they could not be separated.

### Calculating source levels (predictions 1–3)

**Fig 1:**
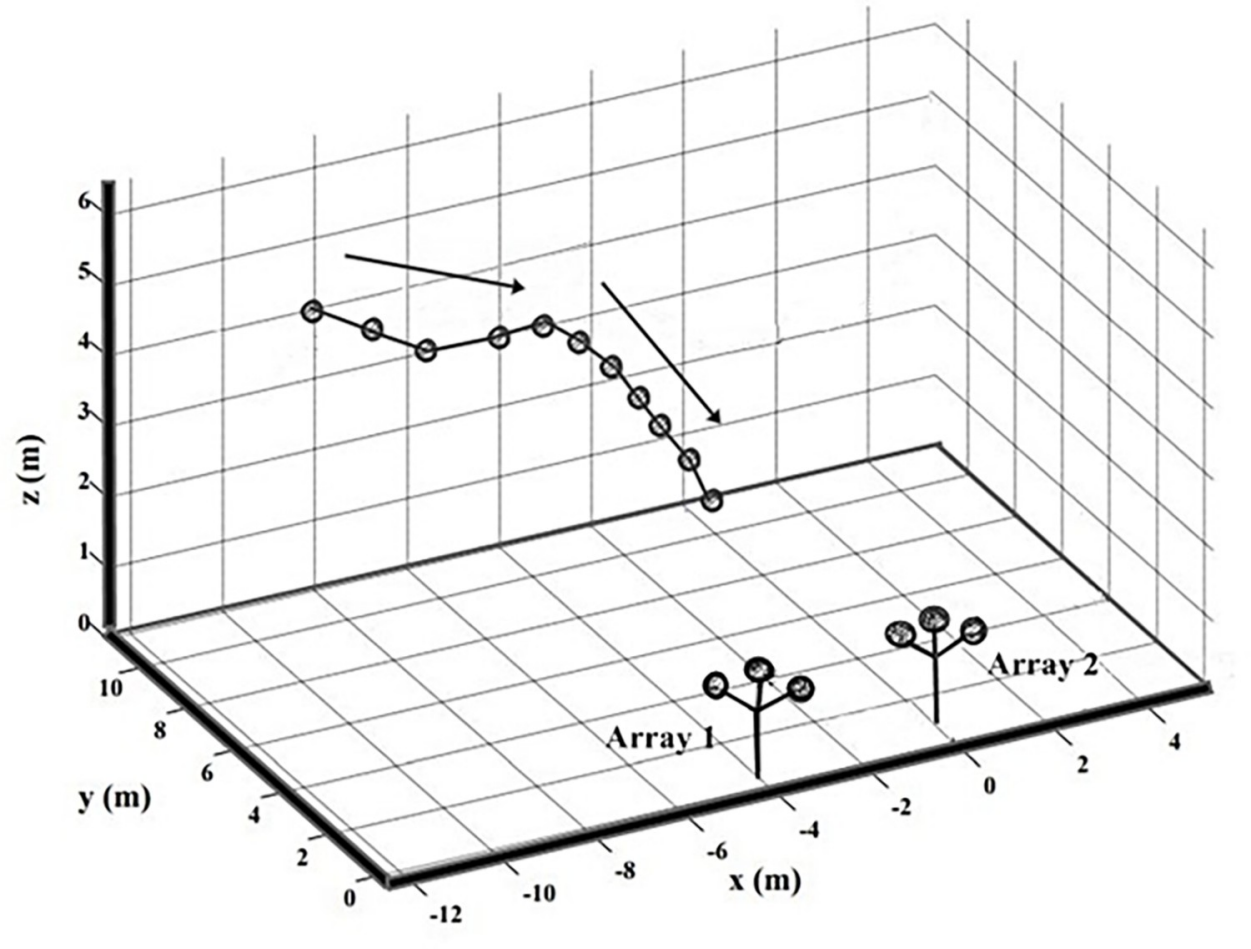
An example of a three-dimensional flight path acquired by acoustic tracking. The illustrated flight path was reconstructed from the echolocation pulse sequences of *Rhinolophus capensis* as it emerged from Wondergat Cave in the desert biome. Circles indicate echolocation pulses and arrows indicate the bat’s direction of travel towards the microphones. The Image was edited in PhotoScape (MOOII Tech, version 3.7, Korea).

Calculation of the source levels (SLs) (sound pressure level of the bats echolocation pulses at a predefined distance of 10 cm in front of the bat in the current study, in decibels [dB]) required that the position of the bat in relation to each microphone be known. Microphone array analysis calculates SL by reconstructing bats flight path in three dimensions in relation to each array [46–48] (Fig 1). Flight paths were re-constructed in Matlab (Mathworks, Version 2013, Natick, United States) following a script used in Holderied and von Helversen [48]. Individual flight paths were only constructed from search phase sequences, even when feeding buzzes were present, i.e. in recordings from foraging areas feeding buzzes were not included in the analysis. Feeding buzzes were distinguished as a sequence of pulses towards the end of a train of pulses where the bat increased its duty cycle to around 90% [49]. We also analysed passes where only one individual was recorded flying in the area covered by the microphone array. From a processing perspective this facilitated trajectory generation by avoiding call-assignation ambiguities, and experimentally this minimized potential behavioural bias created by the presence of nearby conspecifics.

Acoustic flight path reconstructions were based on the differences in the Times of Arrival (TOA) of each pulse at each of the microphones as the bats flew towards the arrays [48,50,51]. TOA differences were calculated using cross-correlation analysis, which measures the similarity between two signals as a function of their relative time delay. Certain pulse designs produce clearer i.e. narrower and less error-prone cross-correlation results [52]. Short broadband FM pulses, with steep frequency modulation are best suited for cross-correlation techniques in contrast to constant frequency signals, that preclude unambiguous measurement of differences in arrival times at the microphones in the array [52–55]. The TOA of the pulses at each of the eight microphones was therefore based on the terminal frequency-modulated component of the echolocation pulses of *R*. *capensis*. The voltage generated by each pulse at the centre microphones of two arrays were converted to recorded dB SPL (taking propagation losses into account) and then converted back to SLs calculated at 10 cm from the bat (calculated for each pulse in a flight path) (Matlab script, ©Holderied). SLs were corrected for both atmospheric attenuation (absorption losses that were dependent on temperature, humidity, and atmospheric pressure) and spreading losses (a decrease in sound pressure levels as a sound propagates away from a source) [9,56,57] based on the distance of the bat to the microphone and the recorded frequency of each pulse [48]. It is important to note that beam width of the emitted call also affects sonar distance by focusing the available energy in one direction or radiating it in many [58]. Techniques to study beam width in HDC bats are new and yet to be applied in field studies. Our field study was also designed to measure accurate SLs of HDC bats rather than measuring their beam width. We minimized the potential bias resulting from off-axis recordings in the following ways. In linear flight bats are likely to point the beam towards their direction of travel [59]. So we chose straight sections of the bat’s flight corridor with bats flying towards the recording microphones. We then only analysed the highest calculated SL per trajectory, thereby selecting the call most closely directed at the recording microphone. Furthermore, the high directionality of HDC bat sound emissions means that only calls directed at the arrays will have sufficient acoustic energy for tracking at all microphones, which already drastically reduces the amount of off- axis recordings in our dataset. Dynamic range was calculated for *R*. *capensis* in the different recording areas by subtracting maximum SLs minus minimum SLs (species level: across all the acoustic flight paths recorded). Automatic parameter measurements (Avisoft-SASLab Pro, v5.2, Avisoft Bioacoustics, Glienicke, Germany) were used to measure echolocation pulse frequency.

Prevailing atmospheric conditions (temperature, humidity, air pressure, and wind speed) at the time of recording of each echolocation pulse, for the determination of bat flight paths, were recorded to allow precise calculation of SLs. Atmospheric variables were recorded using a weather station (Wireless Pro Weather Station, Oregon Scientific, Oregon, USA). The weather station was set up within a few metres of the arrays and three metres above the ground, the maximum flight height of *R*. *capensis* [27].

### Calculating detection distances (testing prediction 1)

Maximum detection distances for detecting different sized prey were calculated using the following formula: DT = SL+ TLA + TLS + TS [48,60–62] where DT is the detection threshold; TLA is transmission loss due to absorption; TLS is the transmission loss due to spherical spreading and TS is the target strength. TLA and TLS are functions of distance. TLA was calculated using the National Physical Laboratory online calculator ((http://resource.npl.co.uk/acoustics/techguides/absorption/). The calculator uses echolocation pulse frequency (frequency of the pulse with the greatest SL in each pass), local atmospheric conditions (temperature, humidity, and atmospheric pressure), and target type to calculate TLA for each pass. Detection distances were calculated using maximum SL (dB SPL) for each pass and the corresponding frequency of that echolocation pulse. The auditory threshold (detection threshold) of the bat was assumed to be 20 dB SPL for bats flying under natural conditions [9]. Target strength is the acoustic energy reflected from an ensonified echolocation signal on a target.

Detection distances were calculated for different prey sizes because target strength varies with the size of the object that reflects the impinging pulse and generates the echo. Each size class has its own target strength, which is used to determine the effective distance at which bats detect different sized prey. Target strengths for each category of insects was assigned as follows: small (-65 dB), medium (-50 dB), and large (-40 dB) in accordance with Stilz and Schnitzler [9]. These target strengths were based on size ranges (4 mm—28 mm) of insects given in previous studies for *R*. *capensis* [48,63]. To determine if the size range of prey available to bats in each habitat (and therefore the target strength) matched those given by these studies, two insect light traps were set up at each recording site at night (from dusk till dawn) over the same time frame in which the acoustic recordings were made. Ultraviolet black lights (Sylvania Blacklight FC22W/350BL UV lamp, Massachusetts, United States) and medium sized buckets (volume: 17.68 L) were used. Collected insect samples were stored in 70% ethyl alcohol and kept in a freezer until analysis. Sample specimens from the orders Coleoptera (beetles) and Lepidoptera (moths), common in the diet of *R*. *capensis* in South Africa, were analysed [27]. Body lengths of these insects were used as a measure of size [27]. Measurements of body lengths were taken from the hind-most tip of the abdomen to the forward most part of the head (excluding the antennae). Sizes of smaller insect were measured using a stereo microscope (Leica EZ4 model, St. Gallen, Switzerland) fitted with a calibrated eyepiece graticule [64,65]. Sizes of larger insects were measured using handheld dial calipers with a ± 0.02 mm increment. The range of sizes of insects collected in this study was then compared to those used by Stilz and Schnitzler [9] to calculate different target strengths.

### Calculating contribution of frequency and SL to detection distance (testing prediction 3)

To determine whether pulse frequencies or SLs has the greatest influence on prey detection distances of *R*. *capensis* in each habitat and in which of these habitats the effect was larger (Prediction 3) we compared detection distances calculated using maximum and minimum values for each parameter i.e. SL and frequency. Detection distance was calculated for each acoustic flight path using the atmospheric conditions present at the time of the pass and the maximum and minimum values recorded for that night for one parameter while keeping the second parameter constant (i.e. using the average recorded value for the second parameter for that night). These detection distances were calculated across one night of recording in each habitat. Within the desert biome, the acoustic parameter that had a greater effect on detection distances (frequency or SL) was determined by comparing detection distances calculated using atmospheric conditions of flight paths recorded at Wondergat cave using a) average frequency of desert passes and varied SLs (average SLs of desert bats versus average SLs of fynbos bats) b) the average SLs of desert bats and different frequencies (average frequency of desert bats versus average frequency of fynbos bats) c) the average frequency and SL for each biome (desert and fynbos).

### Clutter index

A laser range finder (Leica Disto S910, Leica, St. Gallen, Switzerland) measured the distance of objects relative to a reference point creating a three dimensional (3D) image of the recording environment. The 3D image was then used to compare the degree of clutter in each recording environment by creating a clutter index (the percent of space occupied by vegetation or objects). To create this index a grid (8 m width × 8 m length × 3 m height) was constructed over the recording area using thin cord. Grid size was determined by the recording area (the maximum area over which flight paths could be constructed) as well as the average maximum height at which *R*. *capensis* flies when foraging [27]. A measurement point was counted for every metre on the 8 m × 8 m grid if vegetation or an object (e.g. fence, rock) was present. For every metre, measurement points were taken at 0.25 m intervals (starting 0.25 m off the ground) up to 3 m high. Percent clutter (i.e. the clutter index) was calculated by dividing the points recorded by the total possible points for that recording area.

### Statistical analyses

Statistical analyses of acoustic data were performed using STATISTICA (StatSoft, Inc., Version 12, Tulsa, USA) with a global level of significance of 5%. Data were log transformed (= log (#, 10)) because of the different scales of measurement of the parameters. Predictions 1 and 2 were tested using Generalised Linear Models (GLMs). GLMs compared response variables (frequencies, SLs, and detection distances) to categorical variables (species, biomes, and recording environments (foraging versus emergence). Each flight path was an independent variable that consisted of values for its acoustic parameters. Detection distances were calculated using maximum SL (dB SPL) for each pass and the corresponding frequency of that echolocation pulse. A GLM and Wilcoxon Matched Pairs were used to test Prediction 3. A GLM compared the detection indices for each biome. The detection index for each biome was calculated by subtracting detection distances using maximum acoustic parameter values minus detection distance using minimum acoustic parameter values. In the GLM the detection index was the response variable and the two biomes the categorical variable. Wilcoxon Matched Pairs Tests compared detection distance using maximum values for each acoustic parameter against detection distances using minimum values for each acoustic parameter within each biome. Within the desert biome, Wilcoxon Matched Pairs Tests tested for differences in detection distances calculated using the average acoustic parameters of either the desert or fynbos biome.

### Ethical statement

Our study followed the guidelines set out in the American Society of Mammalogists for recording, capturing, and handling bats [66,67]. These methods were approved by the University of Cape Town’s Faculty of Science Animal Ethics Committee (2016/v1/DJ) and Biological Safety Committee. Permits were attained for research in both the Eastern Cape (Cape Nature: AAA007-00012-0052) and Northern Cape (FAUNA 0013/2016) where the study occurred.

## Results

**Fig 2:**
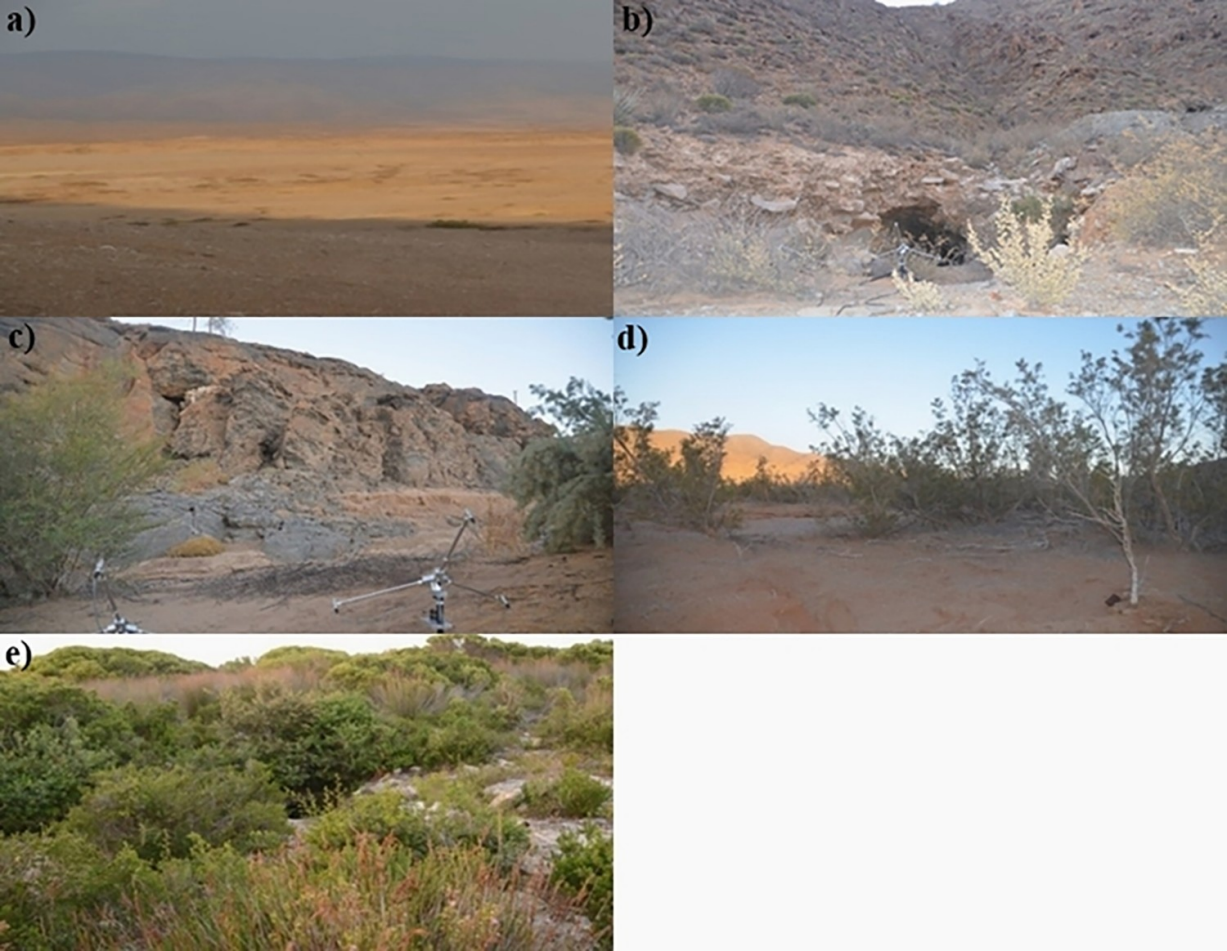
Photographic images taken of recording sites in South Africa of *R*. *capensis* and *R*. *damarensis*. Images are listed from lowest to highest clutter a) Wondergat cave (desert), b) Numeesberg Mineshaft (desert), c) Orange River cave (desert), d) *R*. *capensis* foraging site (desert), e) Hot Hole cave (fynbos).

In the desert biome, 21 flight paths were constructed for *R*. *capensis* during emergence from Wondergat and 19 flight paths were constructed from the desert foraging site. For *R*. *damarensis*, 13 and 19 flight paths were constructed for emergence from Numeesberg mineshaft and the Orange River cave, respectively. In the fynbos biome a combined total of 28 flight paths for *R*. *capensis* were constructed during emergence from Hot Hole cave and whilst the bats were foraging in the area around Hot Hole cave. Prey sampled in both desert and fynbos biomes ranged in size from 4 mm—28 mm (Table 1) and therefore fell into the same size ranges of insect prey reported in previous studies [48,63]. Recording sites ranged in level of clutter (Table 2 and Fig 2). In areas where *R*. *capensis* was recorded, fynbos had the highest degree of clutter (40% clutter) followed by the desert foraging site (24% clutter) and Wondergat cave (7% clutter). In areas where *R*. *damarensis* was recorded, Orange River cave (19% clutter) had the most clutter followed by Numeesberg mineshaft (13% clutter).

**Table 1:** Morphometric data from collected insect samples.

	Sample Size (# insects)	Mean ± SD (mm)	Median (mm)	Range (mm)
**Moths (Lepidoptera)**				
Fynbos	140	13.89 ± 4.33	13.44	5.09–27.78
Desert	153	13.88 ± 3.76	13.44	5.61–23.02
Orange River	38	13.88 ± 3.83	13.55	5.62–20.72
**Beetles (Coleoptera)**				
Fynbos	144	6.24 ± 3.00	13.34	1.91–13.06
Desert	112	8.21 ± 5.46	6.99	2.00–26.74
Orange River	36	5.74 ± 2.34	5.91	2.03–9.82

Mean, standard deviation (SD), median, and range of body lengths (mm) of insects collected from the fynbos biome (8 nights), desert biome (8 nights), and the Orange River site (2 nights).

**Table 2:** Acoustic parameters (average ± SD) of echolocation pulses from *R*. *capensis* and *R*. *damarensis*.

		Clutter Index (%)	N (flight paths)	Peak Frequency (kHz)	Source Levels Average (dB)	Source Levels Range (dB)	Detection Distance (Small) (m)	Detection Distance (Medium) (m)	Detection Distance (Large) (m)
***Rhinolophus capensis***									
**Fynbos**									
	Hot Hole cave	40	28	83.7 ± 0.7	122.3 ± 4.5	117.4–132.9	3.4 ± 0.4	4.9 ± 0.5	6.0 ± 0.5
**Desert**									
	Wondergat cave	7	21	73.2 ± 1.5	127.1 ± 4.4	121.7–133.2	4.1 ± 0.5	5.8 ± 0.6	7.0 ± 0.6
	Foraging area	24	19	74.0 ± 1.2	130.6 ± 3.3	119.5–134.9	4.3 ± 0.3	6.0 ± 0.4	7.3 ± 0.4
***Rhinolophus damarensis***									
	Mineshaft	13	13	82.8 ± 0.6	123.8 ± 8.7	114.1–141.4	3.6 ± 0.8	5.2 ± 0.9	6.3 ± 1.0
	Orange River cave	19	19	83.9 ± 1.5	125.9 ± 6.4	114.9–137.1	3.6 ± 0.6	5.0 ± 0.6	6.1 ± 0.7

Acoustic passes were recorded at different sites in both fynbos and desert habitats. N = sample size.

### Prediction 1 and 2

Echolocation pulses of *R*. *capensis* and *R*. *damarensis* differed significantly in frequency, SL, and detection distances between species and biome (Generalised Linear Model (GLM): F = 239.35, DF = 5, 93, P = 0.00; Table 2).

### Echolocation frequencies (kHz)

Within the desert biome, *R*. *capensis* emitted pulses of similar frequency during emergence and at the nearby foraging area (GLM: F = 64.54, DF = 5, 61, P = 0.00; unequal n HSD, P = 0.11). *R*. *damarensis* recorded emerging/foraging by the Orange River cave emitted pulses of slightly higher frequencies than those of *R*. *damarensis* recorded emerging from Numeesberg mine shaft (GLM: F = 13.61, DF = 5, 26, P = 0.00, unequal n HSD: P = 0.03) (Table 2). Comparing species, *R*. *damarensis* pulses were significantly higher in frequency than *R*. *capensis* pulses recorded in the desert (GLM: F = 293.35, DF = 5, 93, P = 0.00; unequal n HSD: P = 0.00).

Comparing biomes, echolocation pulses of *R*. *capensis* in the desert biome (open habitat) were significantly lower in frequency (unequal n HSD, P = 0.00) than *R*. *capensis* pulses recorded in the fynbos biome (cluttered habitat). *R*. *damarensis* echolocation pulse frequencies were not significantly different to the echolocation pulse frequencies recorded from *R*. *capensis* in the fynbos (unequal n HSD: P = 0.80).

### Source levels (dB)

*R*. *capensis* recorded in the field had a wide dynamic range (maximum—minimum SLs) of 18 dB across individuals and biomes (Table 2). Within the desert biome *R*. *capensis* called louder in the foraging area than during emergence from Wondergat cave (GLM: F = 64.54, DF = 5, 61, P = 0.00; unequal n HSD, P = 0.00; Table 2). Comparing biomes (desert versus fynbos), average SLs for *R*. *capensis* in the desert were higher than those for *R*. *capensis* in the fynbos (unequal n HSD, P = 0.00). In addition, the maximum recorded SLs in the desert was higher than the maximum recorded SLs in the fynbos (Table 2).

The dynamic range of *R*. *damarensis* (28 dB) calculated over both sites was much wider than that in *R*. *capensis* (Table 2). SLs were not significantly different for *R*. *damarensis* between Numeesberg mine shaft and the Orange River cave (GLM: F = 13.61, DF = 5, 26, P = 0.00, unequal n HSD: P = 0.48; Table 2). On average *R*. *damarensis* emitted significantly lower SLs than *R*. *capensis* recorded in the desert (GLM: F = 293.35, DF = 5, 93, P = 0.00; unequal n HSD: P = 0.02) (as a consequence of lower minimum recorded SLs) but not significantly lower SLs than *R*. *capensis* in the fynbos (unequal n HSD: P = 0.16) biome (Table 2). However, maximum recorded SLs for *R*. *damarensis* (141 dB) were substantially higher than those recorded for *R*. *capensis* in both biomes (Table 2).

### Prey detection distances (m)

Within the desert biome (during emergence versus at a nearby foraging area), there was no difference in detection distances for *R*. *capensis* between roost emergence and the foraging site for all prey sizes (GLM: F = 64.54, DF = 5, 61, P = 0.00; small, medium, and large: unequal n HSD, P > 0.05) (Table 2). Comparing biomes (desert versus fynbos), a combination of lower frequency and higher SL pulses yielded significantly longer detection distances (small, medium, and large: unequal n HSD, P < 0.05) for *R*. *capensis* in the desert than in the fynbos (Table 2).

Within the desert biome no significant differences in detection distances were found between *R*. *damarensis* recorded at Numeesberg mine shaft and the Orange River cave (GLM: F = 13.61, DF = 5,26, P = 0.00, small, medium, and large: unequal n HSD, P > 0.05). *R*. *damarensis* had lower average detection distances than *R*. *capensis* in the desert (GLM: F = 293.35, DF = 5, 93; P = 0.00 small, medium, large: unequal n HSD: p < 0.05) but not in the fynbos biome (unequal n HSD: p > 0.05) (Table 2). However, *R*. *damarensis* had higher maximum detection distances (5.3 m, 7.0 m, 8.2 m) (because of higher maximum SLs) than both desert (4.8 m, 6.6 m, 7.9 m) and fynbos *R*. *capensis* (4.1 m, 5.6 m, 6.8 m).

### Relative contributions of frequency and SLs to detection distances (prediction 3)

Within biomes, SLs had a greater influence on detection distances of *R*. *capensis* than frequencies. In each habitat differences in prey detection distances calculated for R. capensis using both maximum and minimum (for that respective biome) SLs was greater than differences in prey detection distances calculated when using maximum and minimum recorded frequencies (Wilcoxon Matched Pairs Test: fynbos: Z = 4.62, P = 0.00, N = 28; desert: Z = 3.82, P = 0.00, N = 19) (Table 3). This effect (differences in prey detection distances when using either maximum or minimum recorded SLs) was larger for the fynbos biome than the desert (GLM: F = 3939, df = 15, 182, p = 0.00; unequal n HSD, p = 0.00) (Table 3). Differences in average recorded SLs between biomes (Table 4A) contributed to greater differences in prey detection within the desert biome than differences in recorded average frequencies (Table 4B; Friedman ANOVA: χ2 = 168, N = 21, P = 0.00). The greatest differences in detection distance were found when comparing detection distances calculated using both average frequencies and SLs of each biome (Table 4C; Friedman ANOVA: χ2 = 168, N = 21, P = 0.00).

**Table 3:** Differences (DIFF) (average ± SD) in calculated detection distances (DD) of small (S), medium (M) and large (L) prey of *R*. *capensis* (in both fynbos and desert biomes).

	N	PF Min (kHz)	PF Max (kHz)	SL Min (dB)	SL Max (dB)	DIFF DD (S) (SL) (m)	DIFF DD (M) (SL) (m)	DIFF DD (L) (SL) (m)	DIFF DD (S) (PF) (m)	DIFF DD (M) (PF) (m)	DIFF DD (L) PF (m)
**Biome**											
Fynbos	28	82	85	117.4	132.9	2.30 ± 0.68	1.93 ± 0.04	2.05 ± 0.00	0.03 ± 0.04	0.05 ± 0.06	0.06 ± 0.08
Desert	19	72	75	123.3	134.9	1.39 ± 0.00	1.52 ± 0.00	1.64 ± 0.00	0.11 ± 0.00	0.16 ± 0.00	0.21 ± 0.00

Differences were calculated when using the maximum and minimum values of one acoustic parameter (either SL = source level or PF = peak frequency), the average of the second acoustic parameter (either SL or PF), and the atmospheric conditions experienced for each site on a single given night (N = number of acoustic flight paths).

**Table 4:** A comparison of the differences in detection distances (average ± SD) of echolocation passes (n = 21 acoustic flight paths) when using different frequencies and SLs from the desert and fynbos biomes. Detection distances were calculated using atmospheric conditions of flight paths recorded in the desert at Wondergat cave with a) average desert frequency with either average SL from the desert and fynbos biome b) average desert SL with average frequency from the desert and fynbos biome c) the average frequency and SL for each biome.

		Detection Distance (Small) (m)	Detection Distance (Medium) (m)	Detection Distance (Large) (m)
**(a)**	**Average Frequency Desert Biome (73khz)**			
	Desert SL (127 dB)	4.92 ± 0.01	6.22 ± 0.02	7.64 ± 0.03
	Fynbos SL (122 dB)	4.32 ± 0.01	5.55 ± 0.02	6.92 ± 0.02
	Difference	0.60 ± 0.00	0.67 ± 0.00	0.72 ± 0.00
**(b)**	**Average SL Desert Biome (127 dB)**			
	Desert Frequency (73kHz)	4.92 ± 0.01	6.22 ± 0.02	7.64 ± 0.03
	Fynbos Frequency (84kHz)	4.62 ± 0.01	5.80 ± 0.02	7.07 ± 0.03
	Difference	0.30 ± 0.00	0.43 ± 0.01	0.57 ± 0.01
**(c)**	**Average Frequency and SL for Desert and Fynbos Biomes**			
	Desert averages: Frequency 73kHz, SL 127 dB	4.92 ± 0.01	6.22 ± 0.02	7.64 ± 0.03
	Fynbos averages: Frequency 84 kHz, SL 122 dB	4.08 ± 0.01	5.20 ± 0.02	6.42 ± 0.02
	Difference	0.84 ± 0.00	1.02 ± 0.00	1.22 ± 0.01

## Discussion

The results of this study suggest that the use of low frequency echolocation pulses resulted in longer detection distances in *R*. *capensis* (as proposed by the FHH) but low frequencies may not be a prerequisite for successful foraging in open biomes. *R*. *capensis* in the desert had longer detection distances than *R*. *capensis* in the fynbos (Prediction 1) because of both lower frequency and higher source level pulses. Lower frequency, higher SL pulses travel further than higher frequency, lower SL pulses, probably allowing *R*. *capensis* in the desert to search greater volumes of open space in the desert biome more efficiently. Higher SLs used by *R*. *capensis* in the desert had a greater contribution to observed differences in detection distances between biomes than frequency (Prediction 3). In addition, on average, *R*. *damarensis* did not compensate for higher frequencies with higher SLs (Prediction 2) resulting in shorter average detection distances than *R*. *capensis* in the desert but not the fynbos (Prediction 1). These results suggest that both predictions were not supported and that the lower frequencies used by *R*. *capensis* in the desert may not be a consequence of selection for longer detection distances. However, a few measurements of SLs for *R*. *damarensis* were the highest recorded and resulted in the longest prey detection distances recorded in this study. The rare use of such high SLs by *R*. *damarensis* in this study, despite the resultant increase in detection distances, may be a consequence of the energetic costs associated with higher SLs [68]. Nevertheless, a combination of both SLs and frequencies produced the greatest differences in detection distances in *R*. *capensis* between biomes. Therefore, the possibility that lower frequency pulses were, at least in part, selected because they allowed longer detection distance in *R*. *capensis* in the desert cannot be excluded.

According to our modified version of the Foraging Habitat Hypothesis (FHH) the proposed ecological significance of lower frequency echolocation pulses in clutter forager specialists (HDC bats) in open habitats (desert) is that lower frequencies increase detection distances [10]. In this study, lower frequency echolocation pulses in *R*. *capensis* in the desert resulted in longer detection distances than *R*. *capensis* in the fynbos. However, *R*. *damarensis*, the heterospecific species that inhabits the same desert habitat as *R*. *capensis* but uses a higher pulse frequency, had shorter average detection distances to *R*. *capensis* in the desert (but not fynbos). Contrary to Prediction 2 they did not compensate for these lower pulse frequencies with higher average SLs. This is contrary to the findings of Surlykke and Kalko [30] that bats within local assemblages compensated for frequency-dependent losses in sound propagation by using higher SLs to achieve comparable prey detection distances. An important caveat to our results were the few high SLs found in *R*. *damarensis*, recorded outside both cave entrances, that produced longer detection distances than those of both fynbos–and desert–inhabiting *R*. *capensis*. These few data points from *R*. *damarensis* might alternatively provide support for Surlykke and Kalko [30] findings as well as the FHH but more research is required to definitively test this, and the effect that energetic costs have on echolocation SLs of bats. In addition to frequency and SL, beam width has also been shown to be a contributing factor to sonar distance [58,59]. In this study we were unable to measure beam width with our microphone array design.

Recent findings by Currie *et al*. [68] have shown that above 130 dB SPL the metabolic cost of sound production is extremely high. The recorded SLs for both *R*. *capensis* and *R*. *damarensis* fell in the range emitted by free-ranging LDC echolocators (120 dB—140 dB) [29,30,48] despite marked differences in echolocation pulse frequency between the different species. Our results and those from other studies suggest that SLs have a large impact on detection distances of bats [30,68]. Currie *et al*. [68] showed that for every dB increase in SL beyond 120 dB SPL *Pipistrellus nathusii* could detect an insect an extra 15 cm away. In our study, SL contributed more to differences in detection distance between biomes than frequency. In addition, when *R*. *damarensis* did emit higher SLs they were able to achieve longer detection distances than desert-inhabiting *R*. *capensis* despite having higher frequencies. However, the benefits of increased SLs (i.e. longer detection distances) are sometimes outweighed by the costs. Refraining from emitting high SLs when the costs of such high emissions does not justify the benefits (such as in high clutter or where prey capture is unlikely) could optimize energetic costs. This is evident in the lower SLs recorded in laboratory versus field environments [30,33–35,48,69] as well as during emergence versus foraging, when prey capture is more likely (our study). It is also possible that these energetic constraints limit bats from constantly emitting pulses with such high SLs. High energy costs could explain the higher maximum, but not average SLs recorded in this study for *R*. *damarensis* compared to desert-inhabiting *R*. *capensis*.

In *R*. *capensis* the evolution of lower frequency pulses combined with the use of higher SLs may allow detection distances to be maximized in the open desert while keeping energy costs low. Mutavhatsindi [32] found similar findings in two species of LDC bats with different foraging strategies and habitats (open air versus clutter). Jacobs *et al*. [70] showed that the frequencies used by *R*. *capensis* at De Hoop are audible to common moths [32,70]. Therefore, in addition to the conservation of energy, using lower SL pulses could minimize the chances of being detected by prey. However, if a combination of SL and frequency is optimal in desert bats then why has *R*. *damarensis* not followed the same evolutionary trajectory as *R*. *capensis* (i.e. evolution of lower frequency pulses). The evolution of lower frequency pulses in *R*. *capensis* in the desert may have therefore evolved for additional purposes other than an increase in detection distances.

The co-inhabitance of two similar species (*R*. *capensis* and *R*. *damarensis*) occupying the same desert environment but using different echolocation pulse frequencies suggests an alternative explanation of why *R*. *capensis* in the desert uses frequencies below 82 kHz. A frequency which deviates more than any other population of *R*. *capensis* from the allometric relationship between body size and pulse frequency for this species [27]. The Acoustic Communication Hypothesis (ACH) [27,40,71] proposes that *R*. *capensis* uses lower frequencies to prevent acoustic overlap with *R*. *damarensis* maintaining effective intraspecific communication. In habituation-dishabituation experiments Bastian and Jacobs [40] found that *R*. *capensis* were able to discriminate between their own pulses and those of *R*. *damarensis*. However, it is important to note that the closest roosts (Wondergat Cave and Numeesberg Mine Shaft) of these species in the desert are 15.8 km apart and their foraging areas might not overlap. Whether they are syntonic, as required by the ACH, is therefore unknown. If the ACH is valid (i.e. differences in frequency are required for species discrimination) then lower frequency pulses in the desert would not only offer longer detection distance, without excess energy expenditure, but also enable unambiguous intra–specific communication.

Frequency has also been shown to be related to prey composition as proposed by the Prey Detection Hypothesis [63,72–74]. In *R*. *capensis* differences in frequency between biomes are not likely due to differences in dietary composition. Insects sampled in this study fell within the same size range giving them equal target strengths [9,72] in both biomes making it unlikely that there is selection pressure from different sized insects. The Sensory Drive Hypothesis (SDH) proposes that climate-induced differences in atmospheric attenuation selects for lower frequency echolocation pulses [75]. The SDH hypothesis was used to explain why *Rhinolophus* bats inhabiting dry habitats produce pulses with higher frequencies than those found occupying humid habitats [12,19,71]. Some studies in HDC bats have demonstrated a complex relationship between frequency and other climatic variables (e.g. atmospheric pressure, temperature, altitude) [12,19,76]. For example, in arid but not mesic habitats [12,19] temperature was found to have the overriding influence on atmospheric attenuation which could explain why in *R*. *capensis* no correlation between humidity and frequency was found between biomes [24]. The pulse frequencies and detection distances used by *R*. *capensis* in both desert and fynbos biomes in this study contradict the SDH. According to this hypothesis, pulses with lower frequencies and therefore longer detection distances should occur in the fynbos environment because it experiences hotter, more humid average nightly conditions (13.90°C, 84%) than the desert (12.05°C, 73%) (South African Weather Service). The SDH hypothesis may explain variation in pulse frequencies used by other rhinolophid species [12,19,76] but it does not appear to explain variation in the observed pulse frequencies and SLs of *R*. *capensis*.

In conclusion, in small HDC bats lower frequency pulses do offer the conferred advantage of longer detection distances without having to exert excess energy expenditure into calling above 130 dB (a decibel range that is associated with a significant increase in energy expenditure in small bats) [68]. However these lower frequencies might not be a prerequisite for successful foraging in open environments suggesting that lower frequency pulses could have evolved in *R*. *capensis* in the desert for additional purposes (such as intraspecific communication). Our study provides the first measurements of SLs and detection distances used by HDC bats in different biomes in the field. These measurements proved vital to improving our understanding of the ecological significance of frequency differences of clutter forager specialist living in different environments.
